# Microbial Risk Factors of Cardiovascular and Cerebrovascular Diseases: Potential Therapeutical Options

**DOI:** 10.2174/1874205X00802010020

**Published:** 2008-05-07

**Authors:** Mohammed Abdalla Abbas, Albrecht Guenther, Sebastiano Galantucci, Gharib Fawi, Giancarlo Comi, Joseph Kwan, Francesco Corea

**Affiliations:** a Neurology Department, Sohag Faculty of Medicine, Sohag University, Sohag, Egypt; b Istituto di Neurologia Sperimentale (INSPE), Istituto di Ricovero e Cura a Carattere Scientifico (IRCCS) San Raffaele, Milano, Neurologia, Dimer, Via Olgettina, 48, 20132, Milano, Italy; c Department of Neurology, Friedrich-Schiller-University, Jena, Erlanger Allee 101, 07747 Jena, Germany; d Department of Medicine for the Elderly, Royal Bournemouth Hospital, Castle Lane East, Bournemouth, BH7 7DW, UK

**Keywords:** Stroke, risk factors, infection, Helicobacter pylori, Chlamydia pneumoniae, Cytomegalovirus, odontopathogens, atherosclerosis, therapy, antibiotics

## Abstract

Infection and inflammation may have a crucial role in the pathogenesis of atherosclerosis. This hypothesis is supported by an increasing number of reports on the interaction between chronic infection, inflammation, and atherogenesis. Assessment of serological and inflammatory markers of infection may be useful adjuncts in identifying those patients who are at a higher risk of developing vascular events, and in whom more aggressive treatments might be warranted.

## INTRODUCTION

Cerebrovascular disease (CVD) is the most common life threatening neurological condition, and is a major contributor to morbidity, functional disability and mortality worldwide.Recent advances in the treatment of acute ischemic stroke offer hope in reducing its devastating effects, but these therapies are only offered to a small fraction of stroke patients. Hence, effective prevention of recurrence, mortality and disability after the initial stroke is especially important [1]. The recognition of the role of inflammation in the pathogenesis, progression and clinical manifestation of atherosclerosis has resulted in a new generation of vascular research. Indeed, the first cell types to be found within the arterial intima in early atherosclerotic lesions are lymphoid cells, macrophages and smooth muscle cells [2,3]. Infections with organisms such as Helicobacter pylori (HP), Chlamydia pneumoniae (CP), Cytomegalovirus (CMV) and odontopathogens, are potentially treatable emerging atherosclerotic risk factors [4]. An increasing number of sero-epidemiological, pathological, immunological and pharmacotherapeutic studies suggest a possible link between these organisms and atherosclerosis, arterial thrombosis and plaque instability.

## HELICOBACTER PYLORI

HP is a gram-negative organism that causes chronic active gastric inflammation, which is probably life-long unless eradicated by antibiotics. HP has been found to be associated

with extra-digestive diseases, in particular, vascular disorders (e.g. ischemic heart disease, primary Raynaud’s phenomenon, ischemic stroke) and autoimmune disorders (e.g. Henoch-Schönlein purpura, Sjögren's syndrome). Many case-control studies have reported a significant association between HP seropositivity and coronary heart disease (CHD) and ischemic electrocardiographic changes, independent of conventional risk factors and socioeconomic factors [5,6].

### Possible Pathological Mechanisms

- Chronic HP infection can cause a low grade chronic inflammatory response, as indicated by raised serological markers including fibrinogen, leukocyte count, and C-reactive protein (CRP) [5].
- Free radical formation may be important as levels of antioxidants have been shown to be decreased in subjects with HP infection [7]; this could result in lipid peroxidation.
- HP infection produces 60 kDa heat shock proteins, which have a high degree of sequence homology with human 60 kDa heat shock proteins; presence of cross-reacting antibodies to heat shock proteins have been shown to be a risk factor for carotid atherosclerosis [8].
- Platelet activation and aggregation have been detected in patients infected with HP [9].
- Hyperhomocysteinemia may possibly be indirectly related to HP infection because chronic gastritis caused by HP infection can lead to vitamin and folate deficiency, which in turn result in problems with methylation by 5-methyl-tetrahydrofolic acid and an accumulation of homocysteine in susceptible patients [10]. Homocysteine is toxic to endothelial cells and results in vascular lesions and ischemic insults.

## CHLAMYDIA PNEUMONIAE

CP is an intracellular gram-negative bacterium that commonly causes respiratory infections in all age groups. Humoral antibodies against CP have been found in more than half of the adult population [11]. IgA antibody may be a more reliable than IgG as a marker for chronic chlamydial infection [11]. A number of cross-sectional and prospective studies have demonstrated an association between high levels of antibody against CP and CHD [12]. Results from many other studies also suggest a role for CP in carotid artery thrombosis [13] and CVD [14-19].

### Possible Pathological Mechanisms

#### Local Effects

CP has been proposed to affect and contribute to the mechanisms involved in atherosclerotic disease process. As a prerequisite, the pathogenic organism must gain entry into the cells of the arterial wall. It is hypothesized that CP may be phagocytosed by alveolar macrophages in the lung and transported by the blood to the subendothelial region through the injured arterial endothelium [20]. Macrophages infected with CP may promote the inflammatory process of atherosclerosis by inducing the production of cytokines and lipoproteins[20], and degenerating into foam cells . CP DNA has been detected in atherosclerotic tissues at different sites of the vascular system.

#### Systemic Effects

- CP can induce an inflammatory response, as indicated by raised CRP, leukocyte count, IL-6, IL-8, tumour necrosis factor α (TNF α), and expression of tissue factor (factor VIIa antigen) [21,22].
- CP can secrete endotoxin and cause activation of monocytes, monocyte integrins and monocyte-derived macrophages [23,24].
- CP infection can activate the nuclear factor for the expression of immunoglobulin κ light chain in the B-lymphocytes pathway (nuclear factor-kappa B)^44^.
- There may be immunological reaction against myosin filaments of carotid artery wall smooth muscle cells due to antigenic mimicry between heavy chains of myosin filaments and specific antigens (40, 42, 52, 54, 60, 75, 98 kDa) presented on CP outer membrane [25].
- Antibodies to chlamydial HSP 60 can cause endothelial cytotoxicity [24]. There is also an antigenic mimicry between human and chlamydial HSP 65; one prospective population-based study strongly supports a potential atherogenic role of persistent CP infection due to immune reactions to HSP 65 [26].
- CP seropositivity can be associated with endothelial dysfunction [27], as indicated by elevated levels of soluble endothelial cell adhesion molecules (sCAMs) as markers of atherosclerotic activity [28].
- CP lipopolysaccharides have been found in circulating immune complexes observed in chronically infected patients [14]. These lipopolysaccharides may have deleterious effects on the coagulation system and vascular endothelium.
- Persistent CP infection may be associated with increased fibrinogen [29], platelet accumulation and adhesion [30], thrombin [31], plasminogen activator inhibitor [22], and enhanced activity of hemostatic and pro-coagulant mediators [30].
- CP infection can cause alteration of cholesterol metabolism and lipid oxidation [23], increased triglycerides [32],increased lipoprotein (a) levels [29], increased total cholesterol levels [33]**, **and decreased high-density lipoprotein cholesterol levels [32,33].

## CYTOMEGALOVIRUS

CMV, or Human Herpes Virus 5, is a double-stranded enveloped DNA virus of the Herpesviridae family. The diagnosis of acute CMV infection is usually based on serology. Newer methods of diagnosis include the presence of pp65 antigen in granulocytes from peripheral blood, viral DNA detection by PCR, viral mRNA detection by nucleic acid sequence-based amplification, conventional cell culture, or detection of early antigen fluorescent foci in cultures [34].

The involvement CMV in some cardiovascular conditions is well known. CMV may also be involved in the pathogenesis of vasculitis, and when this occurs within the central nervous system, it can lead to cerebral infarction [35,36]. There is a growing body of knowledge supporting the hypothesis that CMV may be involved in the development of atherosclerosis [37-40]. Several cross-sectional and prospective studies have indicated that high titres of antibodies to CMV may be associated with CHD [12]. High anti-CMV antibody titre not only appears to be associated with active CHD, but also an early predictor of atherosclerosis [41]. Indeed, CMV infection increases the risk of coronary artery restenosis after angioplasty, stent placement or bypass, and accelerates cardiac transplant-associated coronary atherosclerosis [42]. On the other hand, results from a number of *in vivo* and *in vitro* studies may not support this hypothesis [43-47]. CMV infections have also been found to significantly influence the occurrence of cerebrovascular events in patients with baseline asymptomatic carotid lesions [48], or those with diabetes [49].

### Possible Pathological Mechanisms

- CMV can cause local inflammation,as indicated by the presence of CMV DNA in atheromatous plaques^41^. Vessel wall colonization by CMV may result in cytokine induction or antigen stimulation.
- CMV infection can be associated with thrombocytopenia, thrombotic thrombocytopenic purpura [50], and acute arterial and venous thromboembolism [51, 52].
- CMV seropositivity can be associated with endothelial dysfunction and impaired responses to nitric oxide independently of conventional risk factors [53].
- Chemokine production and expression of virally encoded chemokine receptors have been demonstrated in infected smooth muscle cells (SMC), this can lead to proliferation and/or migration of SMC from the vessel media to the intima, and culminate in vessel narrowing [54].
- Further immunological mechanisms may play a role as indicated by a recent study [2] which revealed that, during CMV infection, a subset of antibodies directed against particular viral proteins cross-reacted with normally expressed cell-surface molecules, causing apoptotic cell death of non-stressed endothelial cells through a mechanism of molecular mimicry. Moreover, in atherosclerosis, the stress induced in endothelial cells by the CMV infection (or reactivation from latency) can lead to the expression of HSP 60. Cross-reactive anti-CMV antibodies may also amplify the endothelial damage by binding surface HSP 60 and inducing cytotoxicity.

## ODONTOPATHOGENS

Periodontitis is a chronic infection by oral bacteria that affect the supporting structures of the teeth. Bacterial populations attached to dental surface consist of biofilm communities, sometimes 50-100 cells in thickness and with a bacterial density of up to 10^11 ^Colony Forming Units/mg, forming a very complex ecosystem. This is due, in part, to the non-shedding surface of the teeth, which allows the development of persistent colonization.

During the past two decades, there has been an increasing interest in the impact of oral health on atherosclerosis and subsequent CVD. Exposure to periodontal infection might influence the development of coronary [55], carotid and peripheral vascular disease [56]. Porphyromonas gingivalis and Streptococcus sanguis are major pathogens associated with periodontitis, and research has shown that these infectious agents may influence vascular cell functions by inducing thrombus formation, vascular cell proliferation, apoptosis, and cell death. A mild to moderate association between periodontitis and the development of CHD and ischemic CVD has been observed [57]. One meta-analysis of prospective and retrospective follow-up studies also concluded that periodontal disease may increase the risk of cardiovascular disease by approximately 20%, and the risk of CVD by almost two-fold [57, 58].

### Possible Pathological Mechanisms

- Gram-negative bacterial infection causes an endotoxemia. Research so far suggests that neopterin, which reflects immune activation of monocytes and macrophages, functions as atherogenic effect modifying factor on the effects of endotoxin [59]. It is hypothesized that immune activation *via *induction of endotoxin hyper-responsiveness determines the atherogenic potential of periodontal infections [59].
- Before periodontal treatments, individuals with periodontitis have been found to have higher concentrations of total and low-density lipoprotein cholesterol and triglycerides, and lower concentrations of high-density lipoprotein cholesterol [60].
- Periodontitis can induce a peripheral inflammatory and immune response, as evidenced by elevated concentrations of CRP and IgA antibodies to periodontal pathogens. The prevalence of CVD appears to be highest in patients with periodontitis and co-existing elevated CRP levels, suggesting that periodontitis might increase the risk of vascular disease through activating the systemic inflammatory and immune responses. There may also be a genetic component which has as yet not been elucidated.
- Porphyromonas gingivitis can cause aggregation of human platelets in platelet-rich plasma [61].

## POTENTIAL THERAPEUTIC OPTIONS

There is now some evidence that the microbial risk factors for vascular disease might be treatable. Several preliminary interventional studies have found that antibiotic therapy might improve the prognosis for patients with coronary heart disease [62,63]. Infection of vascular endothelial cells with CP increases the expression of pro-atherogenic cytokines mediated by the transcription factor nuclear factor (NF)-kappaB, and NF-kappaB-mediated gene activation represents a crucial step in the developmental cycle of CP. One possible reason why aspirin is cardio-protective may be that it can exert an anti-chlamydial effect from inhibiting CP-induced NF-kappaB activation [64]. Macrolides, which are effective against Chlamydia pneumoniae, may also have several non-antibiotic (mostly anti-inflammatory) effects which can contribute towards preventing cardio- and cerebrovascular diseases (see Table **1**) [65]. One study found that certain macrolides (roxithromycin and azithromycin) can significantly reduce the levels of IL-8 and monocyte chemotactic protein (MCP)-1, which in turn may inhibit trans-endothelial migration (TEM) of neutrophils and monocytes [66]. In another prospective study, patients receiving roxithromycin were significantly less likely to suffer an acute coronary event at 30 days [67]. Fong *et al.* [68] showed that clarithromycin administration may modify atherosclerotic lesions by reducing the likelihood of detecting CP bacteria within the lesions. Another crucial development is the emergent role of vaccination in preventing vascular diseases. Observational studies have found that vaccination against influenza may be associated with a reduced risk of stroke, myocardial infarction, and all-cause mortality [69]. Infectious diseases during childhood, even those occurring within the first year or two of life, may predispose an individual to CHD [70].

## CONCLUSION

Infection and inflammation may have a crucial role in the pathogenesis of atherosclerosis. Serum markers of HP, CP, CMV and odontopathogens may predict the risk of CVD even after adjusting of traditional risk factors and socioeconomic status [71]. In the future, assessment of serological and inflammatory markers of infection may become useful adjuncts in identifying those patients who are at a higher risk of developing vascular events, and in whom more aggressive treatments might be warranted. However, the effects and cost implications of this treatment strategy are largely unknown. Vaccination before the initial exposure might be a more logical approach, and one such vaccine is currently being evaluated for CP [72]. Despite a lack of trial evidence, it is recommendable that patients with a previous stroke or at high risk of stroke should receive an annual influenza vaccination. Currently, there is insufficient evidence to recommend the routine administration of antibiotics in preventing cardio- and cerebrovascular diseases in daily clinical practice.

## Figures and Tables

**Table 1.** Non-Antibiotic Effects of Macrolides

Improved endothelial function (azithromycin)
Antioxidant effects (erythromycin, roxithromycin)
Decreased von Willebrand factor levels (azithromycin)
Decreased IL 1, 6 and 8 (azithromycin, clarithromycin, erythromycin, roxithromycin)
Decreased TNFα (azithromycin, clarithromycin, erythromycin, roxithromycin)
Decreased granulocyte/monocyte colony-stimulating factor (clarithromycin)
Decreased monocyte chemotactic protein 1 (azithromycin)
Decreased E-selectin (azithromycin)
Decreased CRP (roxithromycin)
